# *Bacillus licheniformis* Alleviates *Clostridium perfringens*-Induced Intestinal Injury in Mice Model by Modulating Inflammation, Apoptosis, and Cecal Microbial–Metabolic Responses

**DOI:** 10.3390/ani15101409

**Published:** 2025-05-13

**Authors:** Yifan Zhong, Meiting Zhang, Haocheng Xu, Xiaorong Yu, Yashi Hu, Yangyi Xu, Xiao Xiao, Caimei Yang

**Affiliations:** College of Animal Science and Technology, College of Veterinary Medicine, Zhejiang Agricultural and Forestry University, Hangzhou 311300, China; yifanzhong@zafu.edu.cn (Y.Z.);

**Keywords:** *Bacillus licheniformis*, *Clostridium perfringens*, probiotic, intestinal inflammation, apoptosis, cecal microbiota, pentose phosphate pathway, feed additive

## Abstract

*Bacillus licheniformis* (*B. licheniformis*) is a probiotic known for its ability to enhance host resistance against pathogenic infections. This study aimed to evaluate the protective effects and underlying mechanisms of *B. licheniformis* in a mouse model challenged with *Clostridium perfringens* (*C. perfringens*). C57BL/6J mice were pretreated with *B. licheniformis* for 21 days before oral infection with *C. perfringens*. The probiotic administration significantly prevented infection-induced weight loss and immune organ enlargement. Serum cytokine analysis revealed that *B. licheniformis* increased anti-inflammatory IL-4 and IL-10 levels while reducing pro-inflammatory IL-1β, IL-6, and TNF-α levels. Histological analysis showed that *B. licheniformis* preserved intestinal morphology and inhibited epithelial cell apoptosis. Moreover, the probiotic mitigated the infection-induced decline in volatile fatty acid (VFA) production. 16S rRNA gene sequencing revealed that *B. licheniformis* reshaped the cecal microbiota, characterized by the increased abundance of *Lachnospiraceae_NK4A136_group*, *Muribaculaceae*, and *Parabacteroides*, and reduced abundance of *Alistipes*. Untargeted metabolomic profiling identified differential metabolites—including D-glucono-1,5-lactone, D-erythrose 4-phosphate, and D-sedoheptulose 7-phosphate—enriched in the pentose phosphate pathway, suggesting a regulatory role in redox homeostasis and host response. Collectively, these results indicate that *B. licheniformis* exerts protective effects against *C. perfringens* infection by modulating inflammation, apoptosis, microbial composition, and metabolic pathways. This work provides new insights into the application of *B. licheniformis* as a functional microbial feed additive in livestock disease prevention.

## Simple Summary

*Clostridium perfringens* is a common intestinal pathogen that threatens animal health and growth performance. This study investigated the protective effects of the probiotic *Bacillus licheniformis* in mice infected with *C. perfringens*. Results showed that *B. licheniformis* alleviated infection-induced weight loss, reduced inflammation, inhibited intestinal cell apoptosis, and improved gut barrier function. Moreover, the probiotic reshaped the cecal microbiota and modulated key metabolic pathways, including short-chain fatty acid production and the pentose phosphate pathway. These findings suggest that *B. licheniformis* may serve as a promising feed additive for improving intestinal health and disease resistance in animals.

## 1. Introduction

The intestinal tract is not only the primary site for nutrient digestion, absorption, and metabolism but also a critical barrier that maintains internal homeostasis. Maintaining the integrity of the intestinal barrier enhances host resistance to pathogenic invasion and limits excessive inflammatory responses. Conversely, the disruption of the intestinal barrier compromises homeostasis, impairs nutrient absorption, and leads to host metabolic dysfunction [1,2,3,4]. Increasing evidence suggests that the gut microbiota and their metabolites play pivotal roles in reinforcing mucosal barriers and protecting the host from pathogen invasion [5,6]. Dysbiosis, characterized by an increased abundance of pathogenic bacteria and reduced microbial diversity, has been associated with impaired barrier function and the development of intestinal inflammation [7,8].

*Clostridium perfringens* (*C. perfringens*) is a Gram-positive, spore-forming, anaerobic bacterium and a major foodborne pathogen. It is widely implicated in intestinal disorders across animal species and is strongly associated with diarrhea and enterotoxemia [9,10]. *C. perfringens* produces a range of toxins that damage intestinal epithelial cells, induce mucosal shedding, and contribute to intestinal dysfunction [11,12]. Animal studies have shown that *C. perfringens* infection elevates pro-inflammatory cytokine levels and disrupts intestinal morphology [13]. In poultry, *C. perfringens* infection has been linked to reduced growth performance, likely due to impaired nutrient absorption resulting from intestinal barrier damage [14,15].

Probiotics are defined as live microorganisms that confer health benefits to the host when administered in adequate amounts [16]. Probiotic supplementation has been shown to inhibit gastrointestinal pathogens, protect the mucosal barrier, regulate microbial balance, and enhance immune function in both humans and animals [17,18]. Among these, *Bacillus licheniformis* (*B. licheniformis*), a spore-forming and environmentally resilient probiotic strain, has demonstrated strong potential for use as a microbial feed additive [19]. Previous studies have reported that dietary *B. licheniformis* supplementation improves growth performance, modulates fatty acid metabolism, and enhances immune function in poultry [20]. In rodent models, *B. licheniformis* has been shown to regulate intestinal morphology, suppress inflammation, and restore microbial balance under stress conditions [21]. Moreover, Bacillus-based probiotics and their fermented products have been shown to reshape intestinal flora and mitigate intestinal dysfunction in animals, including in broiler chickens infected with *C. perfringens* [15]. However, the mechanistic basis underlying the protective effects of *B. licheniformis* against *C. perfringens* infection remains poorly understood [17].

Therefore, in this study, we investigated the protective efficacy and underlying mechanisms of *B. licheniformis* in C57BL/6J mice challenged with *C. perfringens*, with a specific focus on inflammatory modulation, apoptosis inhibition, and gut microbial–metabolic regulation. These findings may provide theoretical support for the application of *B. licheniformis* as a functional microbial feed additive in animal production.

## 2. Materials and Methods

### 2.1. Animals and Experimental Design

This study was conducted in accordance with the guidelines of the Ethics Committee of Zhejiang Agricultural and Forestry University, Hangzhou, China (approval no. ZAFUAC2022003). Thirty-two female C57BL/6J mice (5 weeks old, 17 ± 1 g) were obtained from the Laboratory Animal Center of Hangzhou Medical College (Hangzhou, China). After a 7-day acclimatization period, the mice were randomly assigned into four groups (*n* = 10 per group): control (CON) group, *B. licheniformis* (BL) group, *C. perfringens*-infected (CP) group, and *B. licheniformis* + *C. perfringens* (BL-CP) group.

**Figure 1:**
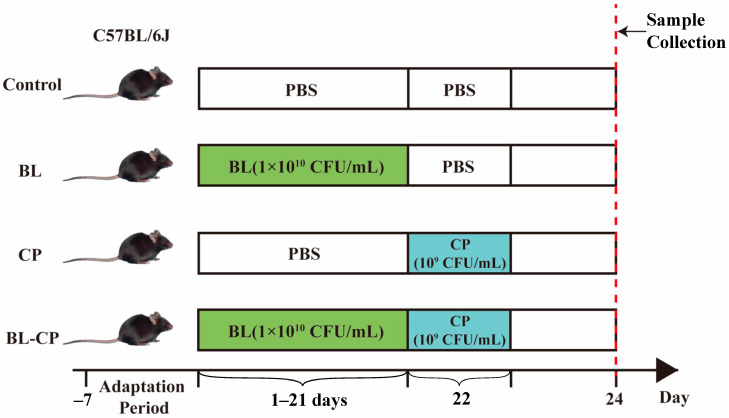
The animal experimental protocol. C57BL/6J mice were assigned to four groups randomly.

Mice in the CON and CP groups received 0.1 mL sterile PBS via oral gavage daily, while those in the BL and BL-CP groups were administered 0.1 mL of *B. licheniformis* HJ0135 (CGMCC 9385) suspension (1 × 10^10^ CFU/mL; Zhejiang Vegamax Biotechnology Co., Ltd., Huzhou, China) daily for 21 consecutive days. On day 22, mice in the CP and BL-CP groups were orally challenged with 0.1 mL of *C. perfringens* ATCC1344 (type A, 1 × 10^9^ CFU/mL). The experimental design is illustrated in Figure 1. Mice were maintained under standard housing conditions with free access to food and water, and bedding was replaced every two days. After 48 h of infection, mice were anesthetized with diethyl ether. Blood was collected via orbital enucleation, and ileum tissue and cecal contents were harvested. Ileum samples (~0.5 cm) were fixed in 4% paraformaldehyde for histological analysis. Remaining tissues and cecal contents were snap-frozen at −80 °C.

### 2.2. Body Weight and Immune Organ Indices

Body weight was recorded every three days throughout the experiment. At euthanasia, liver and spleen were excised, weighed, photographed, and used to calculate the organ indices.

### 2.3. Serum Biochemical Analysis

Blood samples were centrifuged at 3000× *g* for 15 min at 4 °C to obtain serum, which was stored at −80 °C. ELISA kits (Angle Gene Biotechnology Co., Ltd., Nanjing, China) were used to determine serum levels of IL-6, IL-1β, IL-4, IL-10, TNF-α, D-lactic acid (D-LA), and diamine oxidase (DAO), following the manufacturer’s protocols.

### 2.4. Ileum Morphology

Fixed ileum tissues were dehydrated, cleared with xylene, embedded in paraffin, sectioned, and stained with hematoxylin and eosin (H&E). Images were captured using a Nikon microscope (Tokyo, Japan). Villus height (V) and crypt depth (C) were measured using OPTPro 3 software (Chongqing Aote Optical Instrument Co., Ltd., Chongqing, China), and the V/C ratio was calculated.

### 2.5. Western Blot Analysis

Ileum tissues were homogenized in NP-40 lysis buffer containing 1% PMSF (Beyotime, Shanghai, China). Protein concentrations were determined using a BCA Protein Assay Kit (Beyotime, Shanghai, China). Proteins were separated by SDS-PAGE, transferred to PVDF membranes (Merck Millipore, Darmstadt, Germany), blocked in 5% skim milk for 2 h at room temperature, and incubated overnight at 4 °C with primary antibodies: anti-Bax (ET1603-34, Huaan Biotechnology Co., Ltd., Hangzhou, China), anti-Bcl-2 (ET1702-53, Huaan Biotechnology Co., Ltd., Hangzhou, China), and anti-Caspase-3 (ABclonal, Wuhan, China, A11319), all at 1:1000 dilution. Membranes were then incubated with HRP-conjugated goat anti-rabbit IgG (Beyotime, Shanghai, China, A0208, 1:1000) for 2 h at room temperature. Bands were visualized using BeyoECL Plus kit (Beyotime, Shanghai, China) and quantified using Tanon 4600 imaging system. Protein levels were normalized to β-actin.

### 2.6. Volatile Fatty Acid (VFA) Analysis

Cecal contents were mixed with ice-cold distilled water (1:3, *w*/*v*), centrifuged at 12,000× *g* for 10 min at 4 °C. The supernatant was mixed with 25% metaphosphoric acid (5:1, *v*/*v*), incubated on ice for 30 min, centrifuged, and filtered. VFA levels were determined using an Agilent 7890B GC System equipped with a 30 m × 0.25 mm × 0.25 μm capillary column (Agilent Technologies, Santa Clara, CA, USA).

### 2.7. Cecal Microbiota Analysis

Total genomic DNA from cecal contents was extracted using a commercial kit, and DNA integrity was confirmed via agarose gel electrophoresis. The V3–V4 region of the 16S rRNA gene was amplified using primers 515F (5′-CCTAYGGGRBGCASCAG-3′) and 806R (5′-GGACTACNNGGGTATCTAAT-3′). PCR products were purified, recovered, and sequenced on the NovaSeq6000 platform (Novogene, Beijing, China).

Raw sequence data were processed using QIIME2 (v2021.4). Reads were demultiplexed, trimmed, and denoised with DADA2 to generate high-resolution amplicon sequence variants (ASVs). ASVs with <5 reads across all samples were removed. Taxonomic assignment was conducted using the SILVA 138 database.

Alpha diversity metrics including Shannon and Simpson indices were calculated to assess species richness and evenness within samples. Beta diversity was evaluated using principal coordinate analysis (PCoA) and non-metric multidimensional scaling (NMDS) based on Bray–Curtis dissimilarity. Differences in community composition among groups were visualized using ordination plots and statistically tested with PERMANOVA. Differentially abundant taxa at the phylum, family, and genus levels were identified using the MetaStat method with false discovery rate (FDR) correction. Heatmaps were constructed to visualize genus-level changes, focusing on *Lachnospiraceae_NK4A136_group*, *Muribaculaceae*, *Parabacteroides*, and *Alistipes*.

### 2.8. Cecal Metabolome Analysis

Cecal samples (100 mg) were ground in liquid nitrogen and extracted with 80% methanol. Supernatants were prepared and analyzed using a Vanquish UHPLC system coupled with an Orbitrap Q Exactive HF-X mass spectrometer (Thermo Fisher Scientific, Waltham, MA, USA). Chromatographic separation was performed on a Hypesil Gold column (100 × 2.1 mm, 1.9 μm). MS data were acquired in both positive and negative electrospray ionization modes.

Raw MS data were processed using Compound Discoverer (v3.1) for peak alignment, deconvolution, and normalization. Metabolites were annotated against the KEGG database. Principal component analysis (PCA) and partial least squares discriminant analysis (PLS-DA) were performed to explore sample clustering and group separation. Differential metabolites were identified based on VIP > 1.0, *p* < 0.05 (Student’s *t*-test), and fold change ≥ 2 or ≤0.5. Univariate volcano plots and hierarchical clustering heatmaps were used for visualization.

KEGG pathway enrichment analysis was performed using MetaboAnalyst 5.0. Significantly enriched pathways were identified based on hypergeometric testing with adjusted *p*-values. Metabolites including D-glucono-1,5-lactone, D-erythrose 4-phosphate, and D-sedoheptulose 7-phosphate were enriched in the pentose phosphate pathway, indicating metabolic regulation linked to redox balance and intestinal inflammation.

### 2.9. Statistical Analysis

Data were tested for normality using the Shapiro–Wilk test and homogeneity using Levene’s test. Two-way ANOVA followed by Tukey’s HSD test was conducted using JMP Pro 16.0 (SAS Institute, Cary, NC, USA). Results were considered statistically significant at *p* < 0.05. All data are presented as means ± SEM and are visualized using GraphPad Prism 8.0 (GraphPad Software, San Diego, CA, USA).

## 3. Results

### 3.1. B. licheniformis Alleviates Intestinal Injury Caused by C. perfringens Infection

**Figure 2:**
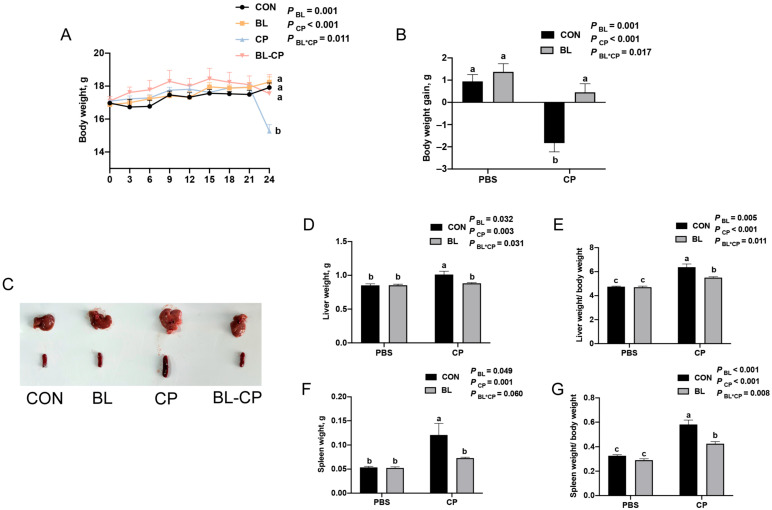
*B. licheniformis* alleviated *C. perfringens* infection. (**A**) Body weight of mice. (**B**) Body weight changes in mice. (**C**) Morphology of liver and spleen. (**D**) Liver weight. (**E**) Liver index. (**F**) Spleen weight. (**G**) Spleen index. Bars with different letters are statistically significant (*p* ˂ 0.05) in different groups.

To investigate the protective effects of *B. licheniformis* against *C. perfringens* infection, C57BL/6J mice were pretreated with *B. licheniformis* for 21 days, followed by oral challenge with *C. perfringens* for 22 days. As shown in Figure 2A,B, bod weight of mice showed no significant difference before challenge mice in the CP group exhibited a significant reduction in body weight compared to the CON group (*p* < 0.05), while *B. licheniformis* pretreatment significantly mitigated this weight loss (*p* < 0.05). Additionally, liver and spleen weights and their respective organ indices were significantly increased after *C. perfringens* infection (Figure 2C–G). These changes were reversed by *B. licheniformis* supplementation, suggesting a protective effect against immune organ hypertrophy induced by infection.

### 3.2. B. licheniformis Reduces Systemic Inflammation Induced by C. perfringens

**Figure 3:**
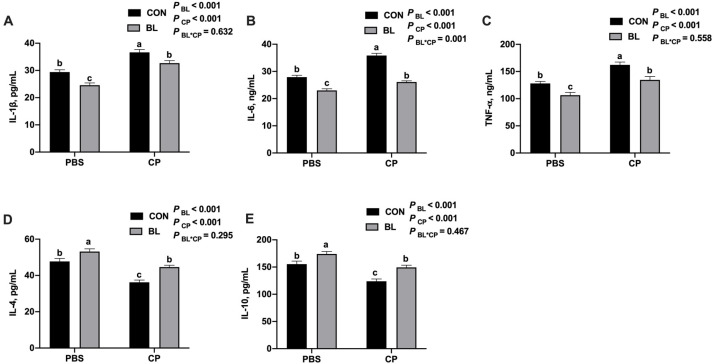
*B. licheniformis* reduced systemic inflammation caused by C. perfringens. (**A**) IL-1β content; (**B**) IL-6 content; (**C**) TNF-α content; (**D**) IL-4 content; (**E**) IL-10 content. Bars with different letters are statistically significant (*p* ˂ 0.05) in different groups.

To assess systemic inflammatory responses, serum cytokine levels were measured. Compared to the CON group, *C. perfringens* infection significantly elevated serum levels of IL-1β, IL-6, and TNF-α, while suppressing IL-4 and IL-10 (*p* < 0.05; Figure 3A–E). Pretreatment with *B. licheniformis* significantly reversed these effects, decreasing pro-inflammatory cytokines and increasing anti-inflammatory cytokines (*p* < 0.05), indicating an anti-inflammatory role of the probiotic.

### 3.3. B. licheniformis Preserves Intestinal Morphology Disrupted by C. perfringens

**Figure 4:**
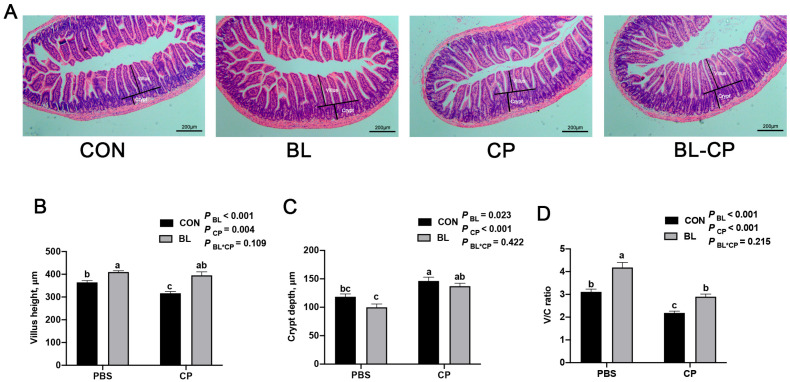
*B. licheniformis* repaired intestinal morphology damaged by *C. perfringens*. (**A**) The ileum morphology of mice. (**B**) The villus length in the ileum of mice. (**C**) The crypt depth in the ileum of mice. (**D**) The ratio of the villus height and crypt depth. Bars with different letters are statistically significant (*p* ˂ 0.05) in different groups.

Histological analysis of ileum sections revealed that *C. perfringens* infection caused villus atrophy, crypt hyperplasia, and disrupted mucosal integrity (Figure 4A). Quantitative measurements showed significant reductions in villus height and V/C ratio, alongside increased crypt depth in the CP group (*p* < 0.05; Figure 4B–D). These histopathological alterations were markedly alleviated in the BL-CP group, suggesting that *B. licheniformis* maintains structural integrity of the intestinal epithelium.

### 3.4. B. licheniformis Improves Intestinal Barrier Function

**Figure 5:**
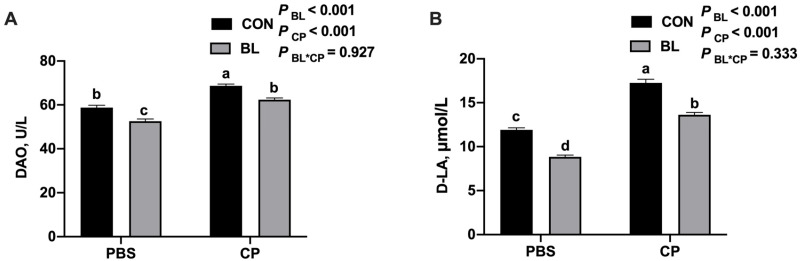
*B. licheniformis* modulated intestinal barrier during *C. perfringens* infection. (**A**) DAO content. (**B**) D-LA content. Bars with different letters are statistically significant (*p* ˂ 0.05) in different groups.

To further evaluate intestinal barrier integrity, serum levels of D-lactic acid (D-LA) and diamine oxidase (DAO) were measured. As shown in Figure 5A,B, *C. perfringens* infection significantly increased both D-LA and DAO levels, which is indicative of intestinal permeability and mucosal damage (*p* < 0.05). *B. licheniformis* pretreatment significantly reduced these biomarkers (*p* < 0.05), supporting its role in maintaining barrier function during infection.

### 3.5. B. licheniformis Inhibits Ileal Epithelial Apoptosis

**Figure 6:**
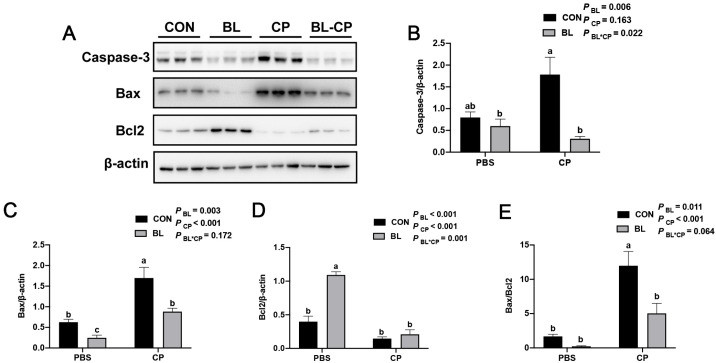
*B. licheniformis* inhibited apoptosis induced by *C. perfringens*. (**A**) Protein expression of Caspase-3, Bax and Bcl-2; (**B**) Caspase-3 protein expression level; (**C**) Bax protein expression level; (**D**) Bcl-2 protein expression level; (**E**) Bax/Bcl2 expression level. Bars with different letters are statistically significant (*p* ˂ 0.05) in different groups (*n* = 3 each).

To determine whether *B. licheniformis* attenuates intestinal apoptosis, we analyzed the expression of apoptosis-related proteins. As shown in Figure 6A–E, *C. perfringens* infection significantly increased the expression of pro-apoptotic proteins Bax and Caspase-3, as well as the Bax/Bcl-2 ratio (*p* < 0.05), and decreased the expression of anti-apoptotic Bcl-2. In contrast, *B. licheniformis* pretreatment downregulated Bax and Caspase-3 levels, upregulated Bcl-2, and significantly reduced the Bax/Bcl-2 ratio (*p* < 0.05), indicating the suppression of epithelial cell apoptosis.

### 3.6. B. licheniformis Modulates Cecal Short-Chain Fatty Acid Production

**Figure 7:**
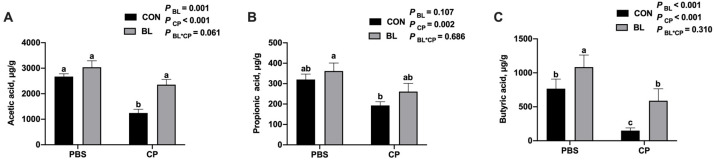
*B. licheniformis* modulated VFAs contents during *C. perfringens* infection. (**A**) Acetic acid content. (**B**) Propionic acid content. (**C**) Butyric acid content. Bars with different letters are statistically significant (*p* ˂ 0.05) in different groups.

To assess microbial metabolic function, volatile fatty acids (VFAs) in the cecal contents were quantified. As shown in Figure 7A–C, *C. perfringens* infection significantly decreased the concentrations of acetic acid and butyric acid (*p* < 0.05), while *B. licheniformis* supplementation restored these levels. Notably, butyric acid levels were significantly elevated in the BL group compared to CON (*p* < 0.05). *C. perfringens* infection significantly reduced the level of propionic acid compared to the control group (*p* < 0.01). Although supplementation with *B. licheniformis* showed a trend toward increasing propionic acid levels in both uninfected and infected mice, the differences were not statistically significant when compared to the respective control groups (*p* = 0.107 for BL vs. CON; *p* = 0.686 for BL-CP vs. CP). These results suggest that *B. licheniformis* modulates SCFA production and maintains microbial fermentation capacity during infection.

### 3.7. B. licheniformis Reshapes Gut Microbial Composition and Diversity

**Figure 8:**
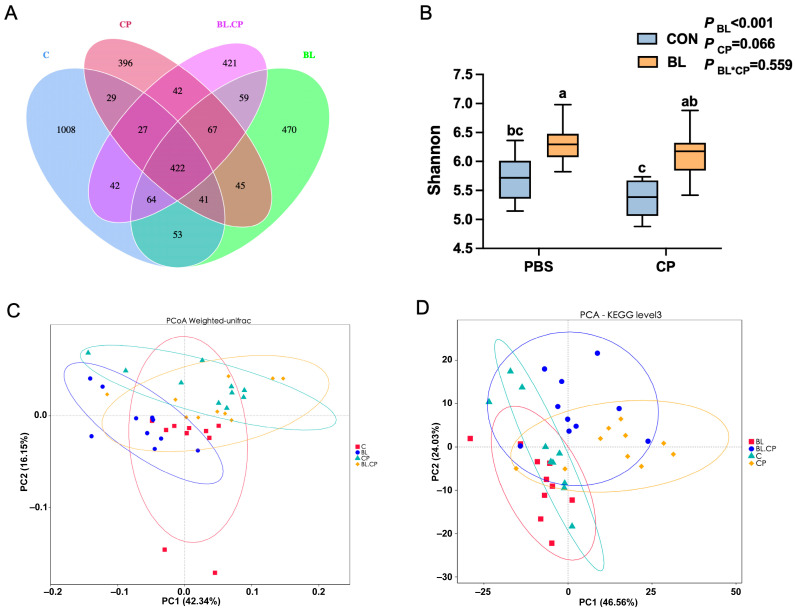
Analysis of the diversity of gut microbiota. (**A**) Venn diagram summarizing the numbers of common and unique ASVs in the microflora community in the cecal in mice. (**B**) Shannon index. (**C**) PCoA analysis. (**D**) PCA Plot of KEGG at level 3. Bars with different letters are statistically significant (*p* ˂ 0.05) in different groups.

To explore how *B. licheniformis* affects gut microbial ecology during infection, 16S rRNA gene sequencing of cecal contents was performed. A total of 422 amplicon sequence variants (ASVs) were shared among the four groups, with CP and BL-CP groups showing markedly reduced richness (Figure 8A). Shannon and Simpson indices showed that *B. licheniformis* pretreatment increased microbial diversity compared to CP (*p* < 0.05; Figure 8B,C).

**Figure 9:**
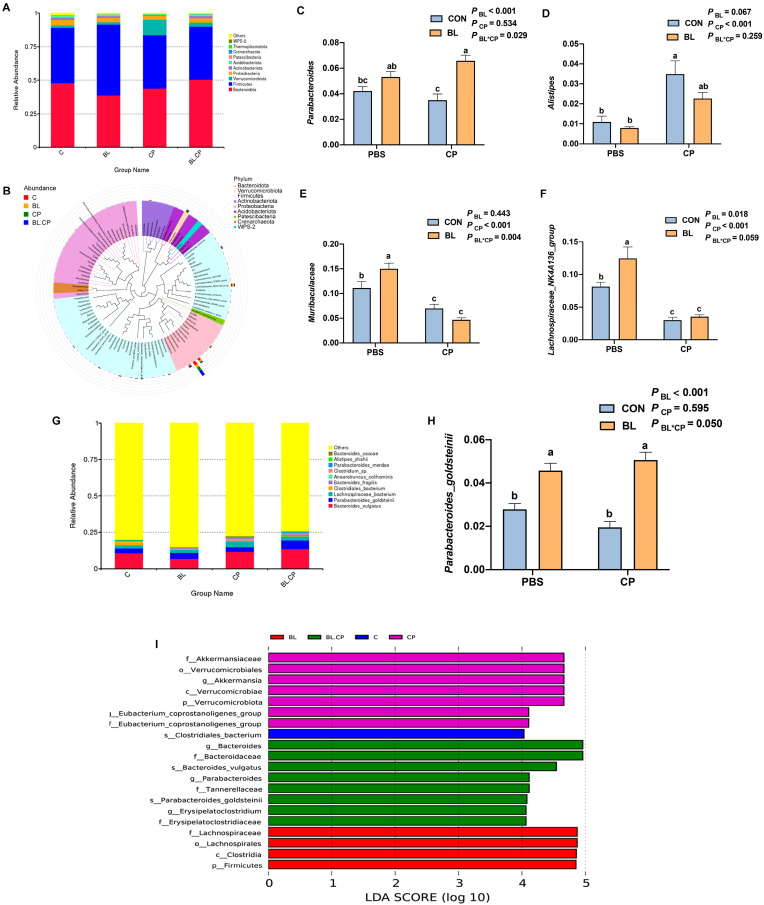
Summary of the microbial community composition in the cecal contents of mice. (**A**) Bar graph of microbial composition at the phylum level. (**B**) Species evolutionary tree at the genus level. (**C**–**F**) Box plot of the significant genera among groups. (**G**) Bar graph of microbial composition at the species level. (**H**) Box plot of significant species among groups. (**I**) The LDA score of bacteria enriched in groups. Bars with different letters are statistically significant (*p* < 0.05) in different groups.

Beta diversity analyses using PCoA and NMDS revealed distinct clustering of microbial communities between BL and CP groups (Figure 8D), indicating significant shifts in microbiota composition. Taxonomic profiling (Table S1) at the phylum level identified Bacteroidota and Firmicutes as dominant phyla (Figure 9A). At the genus level (Table S2), *Lachnospiraceae_NK4A136_group*, *Muribaculaceae*, *Parabacteroides*, and *Alistipes* were dominant (Figure 9B).

*C. perfringens* infection significantly reduced the relative abundance of *Lachnospiraceae_NK4A136_group* and *Muribaculaceae*, while increasing *Alistipes* (*p* < 0.05). *B. licheniformis* reversed these changes and also increased *Parabacteroides* abundance (Figure 9C–F). At the species level, *Parabacteroides*_*goldsteinii* was significantly elevated in the BL group (*p* < 0.05; Figure 9H), indicating species-specific microbiota regulation. The enriched bacteria identified by LEfSe analysis in each group were shown in Figure 9I.

### 3.8. B. licheniformis Alters Cecal Metabolic Profiles During Infection

**Figure 10:**
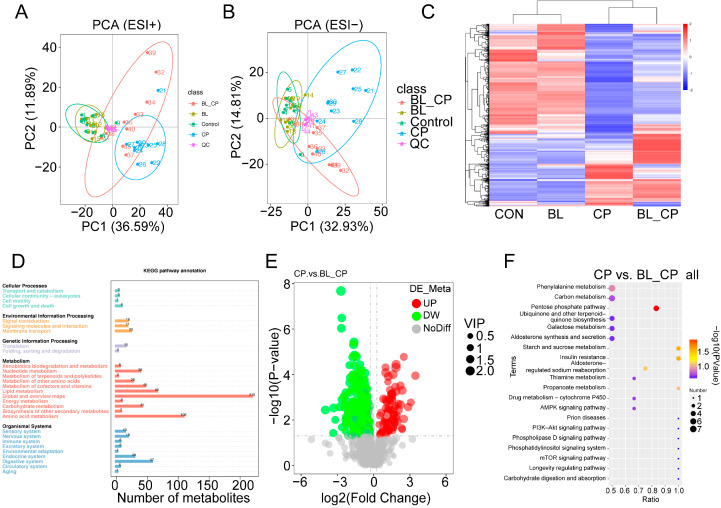
Summary of the metabolomics analysis in the cecal contents of mice. (**A**,**B**) PCA plot of mice’ cecal metabolome. (**C**) Hierarchical clustering analysis of cecal metabolites. (**D**) KEGG pathway classification. (**E**) Volcano plot of metabolites between CP and BL-CP groups. (**F**) Bubble diagram showing the KEGG enrichment analysis.

The metabolic profiles between four groups have been investigated (Tables S3 and S4). Principal component analysis (PCA) revealed the distinct clustering of metabolite profiles among the four treatment groups in both positive and negative ion modes (Figure 10A,B). Hierarchical clustering analysis further showed marked differences in metabolite abundance between groups (Figure 10C). KEGG pathway classification suggested that differential metabolites were primarily involved in lipid metabolism, amino acid metabolism, and carbohydrate metabolism (Figure 10D).

A total of 28 differential metabolites were identified between the CON vs. BL and CP vs. BL-CP groups, mainly classified into fatty acyls, glycerophospholipids, steroids, and steroid derivatives (Table 1). Among them, 16 metabolites were upregulated in BL vs. CON and 15 were upregulated in BL-CP vs. CP. Bubble plot analysis showed that the top enriched pathways included the pentose phosphate pathway (PPP), insulin resistance, and starch/sucrose metabolism (Figure 10E,F).

### 3.9. B. licheniformis Activates the Pentose Phosphate Pathway in the Gut

**Figure 11:**
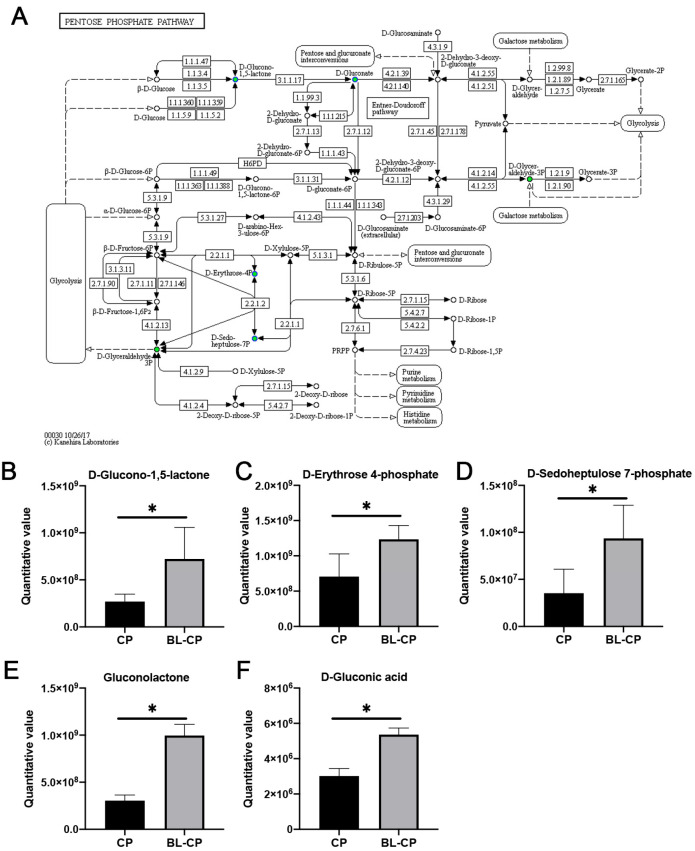
Analysis of pentose phosphate pathway. (**A**) KEGG pathway map of pentose phosphate pathway (map00030). (**B**–**F**) Box plots of the differential metabolites between groups. * represents a significant difference at *p* < 0.05.

KEGG enrichment results revealed that the pentose phosphate pathway (PPP) was significantly enriched in BL-CP mice compared to CP mice (*p* = 0.0101; Figure 11A). Five key metabolites within this pathway—including D-glucono-1,5-lactone, D-erythrose 4-phosphate, D-sedoheptulose 7-phosphate, gluconolactone, and D-gluconic acid—were significantly upregulated in *B. licheniformis*-treated mice (*p* < 0.05; Figure 11B–F). This suggests that *B. licheniformis* promotes redox homeostasis and energy metabolism via the activation of the PPP under pathogenic challenge conditions.

## 4. Discussion

Maintaining intestinal health and functionality is crucial for optimal animal growth and immune performance. However, factors such as bacterial infection, contaminated feed, weaning stress, and environmental changes can compromise intestinal integrity and function [20,22]. *Clostridium perfringens* (*C. perfringens*), a common pathogenic bacterium, has been shown to impair growth, induce intestinal lesions, and cause systemic immune dysregulation in various animals. For instance, in broilers, *C. perfringens* infection reduces nutrient absorption and growth rate, while in mice, it has been associated with decreased body weight, shortened colon length, and enlarged spleen [13,18,21]. Although multiple challenges are sometimes necessary to induce intestinal pathology in certain animal models, consistent with these reports, our preliminary experiments showed that a single-dose infection was sufficient to trigger consistent and measurable outcomes. We observed that *C. perfringens* infection caused significant weight loss and organ enlargement in mice. Consistent with these reports, we observed that *C. perfringens* infection caused significant weight loss and organ enlargement in mice. Importantly, *Bacillus licheniformis* supplementation effectively alleviated these adverse effects, aligning with previous studies in poultry where *B. licheniformis* improved growth and intestinal health during infection [16].

Pathogenic infections such as those caused by *C. perfringens* and *Salmonella Typhimurium* often elicit excessive inflammatory responses [23,24]. Sustained high levels of pro-inflammatory cytokines can trigger systemic inflammation and intestinal injury—a condition referred to as cytokine release syndrome [25]. One of the mechanisms by which *B. licheniformis* exerts its protective effects may involve immunomodulation through cytokine regulation. In line with earlier findings in piglets and IPEC-J2 cells [26], we observed that C. perfringens infection significantly increased the serum levels of IL-1β, IL-6, and TNF-α, while decreasing those of IL-4 and IL-10. These effects were reversed by *B. licheniformis*, supporting its role in immune modulation. Previous reports further confirm this function, demonstrating that *B. licheniformis* reduces pro-inflammatory cytokines in heat-stressed rats [27] and downregulates inflammatory responses in cell lines when combined with Bifidobacterium breve [28]. Thus, our findings suggest that *B. licheniformis* enhances immune resilience by rebalancing cytokine profiles during infection. We acknowledge that the absence of a dose–response design limits the ability to determine the minimal effective dose or optimal concentration range. Although our primary objective was to validate the protective effect of a biologically active dose against *C. perfringens*-induced intestinal injury, future studies should include multiple dose levels to establish a clearer dose–effect relationship and to evaluate the safety and efficacy of *B. licheniformis* HJ0135 in a more comprehensive manner.

Excessive inflammation can lead to epithelial cell apoptosis and the disruption of intestinal structure [29]. The intestinal mucosa plays a dual role in nutrient absorption and immune defense [30], and its functionality depends heavily on morphological integrity, particularly villus height and crypt architecture [10,31,32]. *C. perfringens* has been shown to cause villus atrophy and epithelial damage in poultry [33], and we observed similar damage in mice ileal tissue. *B. licheniformis* significantly preserved villus structure and increased the villus height-to-crypt depth ratio, indicating improved absorptive capacity and mucosal repair, consistent with previous probiotic studies in both stressed and infected mouse models [34,35].

Intestinal permeability is a hallmark of barrier integrity, preventing antigen translocation while supporting nutrient and fluid absorption [36,37]. Serum diamine oxidase (DAO) and D-lactic acid (D-LA) are widely recognized biomarkers of mucosal injury and barrier dysfunction [38,39,40]. In our study, *C. perfringens* infection elevated both DAO and D-LA levels, indicating compromised barrier function, while *B. licheniformis* pretreatment restored these biomarkers to near-normal levels. These findings are consistent with previous work demonstrating the barrier-protective role of probiotics such as *Bifidobacterium lactis* [41]. In the present study, we assessed intestinal barrier integrity by measuring serum levels of diamine oxidase (DAO) and D-lactic acid, both of which are widely recognized biomarkers for epithelial injury and increased permeability in murine models. However, we acknowledge that the complexity of mucosal barrier function cannot be fully captured by only two indicators. Additional biomarkers, such as serum lipopolysaccharide (LPS), Toll-like receptor 4 (TLR4), and tight junction proteins, including MUC2, Occludin, and Zonula Occludens-1 (ZO-1), are also important for a comprehensive evaluation of intestinal barrier status. Although these parameters were not measured in the current study due to experimental scope and resource constraints, future investigations will incorporate these markers to provide a more detailed understanding of the mechanisms by which *B. licheniformis* modulates intestinal barrier function. This will further strengthen the mechanistic insights into the probiotic’s protective role against *C. perfringens*-induced epithelial damage.

Apoptosis is a tightly regulated process essential for tissue homeostasis, primarily governed by intrinsic mitochondrial signaling involving the Bcl-2 protein family [42,43,44]. Disruption in the balance between pro-apoptotic (Bax) and anti-apoptotic (Bcl-2) factors leads to caspase activation and programmed cell death [45,46]. Our results showed that *C. perfringens* infection elevated Caspase-3 and Bax levels and increased the Bax/Bcl-2 ratio, indicating apoptosis in the ileal mucosa. In contrast, *B. licheniformis* reversed these changes, highlighting its anti-apoptotic potential. Similar findings have been reported for other probiotic strains, including *Lactobacillus plantarum* and *Paenibacillus polymyxa*, in both mice and poultry models [47,48].

Microbial diversity plays a critical role in gut homeostasis and host defense [49]. Short-chain fatty acids (SCFAs), primarily produced by microbial fermentation, are essential for barrier integrity, immune modulation, and pathogen exclusion [50,51,52]. *C. perfringens* infection disrupted microbial composition in our study, reducing beneficial taxa such as *Lachnospiraceae_NK4A136_group* and *Muribaculaceae*, while increasing *Alistipes*, a genus associated with inflammation [52,53]. *B. licheniformis* pretreatment reversed these shifts and increased *Parabacteroides*, a known SCFA producer [54,55]. This was accompanied by increased acetic and butyric acid concentrations, supporting the hypothesis that microbiota modulation underlies the functional benefits of *B. licheniformis*. Previous studies have shown strong correlations between the abundance of *Muribaculaceae* or *Lachnospiraceae* and reduced inflammatory markers such as IL-1β and IL-6 [56,57,58]. Although *B. licheniformis* supplementation did not significantly restore cecal propionic acid levels in *C. perfringens*-infected mice, a modest upward trend was observed. Propionic acid, a key short-chain fatty acid (SCFA), plays an important role in maintaining intestinal homeostasis by modulating immune responses, reducing pro-inflammatory cytokine production, and serving as an energy source for colonocytes. The observed decrease in propionic acid following *C. perfringens* infection is consistent with microbial dysbiosis and impaired fermentation activity. Although the protective effect of *B. licheniformis* on propionic acid production was not statistically significant, the partial recovery may indicate subtle modulatory effects on microbial composition or metabolism that warrant further investigation. Future studies using targeted metabolomics and microbial community profiling may help clarify the role of *B. licheniformis* in regulating specific SCFA pathways under pathogenic stress.

Our metabolomic analysis identified 28 differential metabolites primarily enriched in pathways related to lipid metabolism, steroid biosynthesis, and the pentose phosphate pathway (PPP), indicating that *B. licheniformis* exerts a broad regulatory impact on host metabolic responses. Among these, glycerophospholipids such as phosphatidylcholines and phosphatidylethanolamines are known to play key roles in maintaining membrane integrity, modulating toll-like receptor signaling, and dampening inflammatory cascades through bioactive lipid mediators [59,60], Their alteration in the CP group suggests membrane instability and oxidative stress, which were partially alleviated by *B. licheniformis* intervention. Importantly, *B. licheniformis* significantly upregulated five key metabolites within the PPP—D-glucono-1,5-lactone, D-erythrose 4-phosphate, D-sedoheptulose 7-phosphate, gluconolactone, and D-gluconic acid. The PPP is a central metabolic route for generating NADPH, which is essential for maintaining intracellular redox balance and supporting the biosynthesis of nucleotides and lipids. Enhanced flux through the PPP may contribute to antioxidant defense mechanisms, particularly under inflammatory stress triggered by *C. perfringens*. Gluconolactone, in particular, has been reported to inhibit Caspase-3 activation and enhance the expression of the anti-apoptotic protein Bcl-2 in various stress models [61], consistent with our findings of decreased apoptosis in ileal tissues upon *B. licheniformis* treatment. Additionally, D-erythrose 4-phosphate and D-sedoheptulose 7-phosphate are intermediates that bridge the PPP and glycolysis, linking energy metabolism to immune regulation. Their upregulation may reflect enhanced metabolic flexibility and a shift toward anti-inflammatory phenotypes in the host. These findings suggest that modulation of host metabolism—especially through redox-sensitive pathways like the PPP—represents a key mechanism by which *B. licheniformis* confers protection against *C. perfringens*-induced intestinal injury. The future targeted validation of these metabolic nodes may reveal novel therapeutic targets for probiotic intervention.

From a practical perspective, the findings of this study suggest that *Bacillus licheniformis* HJ0135 holds considerable potential as a prophylactic probiotic to mitigate intestinal damage caused by *Clostridium perfringens* infections. The observed improvements in intestinal barrier function, the modulation of apoptotic pathways, and the restoration of short-chain fatty acid profiles highlight the capacity of this strain to support gut health under pathogenic stress. These results are particularly relevant to livestock industries, where *C. perfringens*-related enteritis poses a significant threat to animal welfare and productivity. While this study was conducted in a mouse model, the mechanisms identified—such as regulation of redox metabolism and epithelial integrity—are conserved across mammalian species, including pigs and poultry. Future studies should focus on validating the efficacy of *B. licheniformis* HJ0135 in production animals under farm-like conditions, including the evaluation of growth performance, disease resistance, and microbiota modulation, to support its use as a functional feed additive in animal agriculture.

## 5. Conclusions

In conclusion, *Bacillus licheniformis* HJ0135 effectively alleviated intestinal damage induced by *Clostridium perfringens* infection in mice by reducing systemic inflammation, suppressing epithelial apoptosis, restoring intestinal morphology, and modulating cecal microbiota composition and metabolic activity. Notably, its ability to upregulate key metabolites in the pentose phosphate pathway (PPP) and partially restore short-chain fatty acid (SCFA) levels suggests a multifaceted mechanism involving immune regulation, oxidative stress mitigation, and metabolic reprogramming. These findings advance current knowledge of host–microbe interactions under enteric pathogen challenge and highlight the strain-specific probiotic potential of *B. licheniformis* HJ0135. Furthermore, the results lay a foundation for future translational studies in production animals, supporting its application as a functional probiotic feed additive to promote intestinal health, enhance disease resilience, and reduce reliance on antibiotics in livestock farming.

## Figures and Tables

**Table 1:** Comparisons of cecal metabolic biomarkers.

Number	Metabolites	Formula	Category	Mass (M/Z)	RT (Min)	CON vs. BL		CP vs. BL-CP	
						Trend	*p* Value	Trend	*p* Value
1	12-Oxo-phytodienoic acid	C18H28O3	Fatty Acyls	293.2114	6.233	up	0.0004	up	0.0223
2	19(R)-hydroxy Prostaglandin A2	C20H30O5	Fatty Acyls	331.1920	6.951	up	0.0011	up	0.0028
3	Docosatrienoic acid	C22H38O2	Fatty Acyls	333.2804	11.139	down	0.0198	down	0.0017
4	10-Undecenoic acid	C11H20O2	Fatty Acyls	185.1539	6.417	down	0.0292	down	0.0001
5	Deoxycorticosterone	C21H30O3	Steroids and steroid derivatives	699.4053	5.368	up	0.0050	up	0.0017
6	7-Ketodeoxycholic acid	C24H38O5	Steroids and steroid derivatives	405.2649	6.586	up	0.0095	down	0.0005
7	17α-Ethynylestradiol	C20H24O2	Steroids and steroid derivatives	297.1853	6.594	up	0.0266	down	0.0021
8	Taurodeoxycholic acid sodium salt	C26H44NNaO6S	Steroids and steroid derivatives	522.2875	8.99	up	0.0287	down	0.0257
9	11-Oxoetiocholanolone	C19H28O3	Steroids and steroid derivatives	287.2006	7.303	up	0.0336	down	0.0057
10	Cortisol	C21H30O5	Steroids and steroid derivatives	363.2171	6.229	up	0.0467	down	0.0358
11	LysoPE 16:0	C21H44NO7P	Glycerophospholipids	452.2792	8.566	up	0.0288	up	0.0033
12	LysoPC 12:1	C20H36NO7P	Glycerophospholipids	432.2153	7.81	down	0.0450	down	0.0000
13	LysoPC 14:0	C22H46NO7P	Glycerophospholipids	512.2999	8.565	up	0.0038	up	0.0004
14	LPG 4:0	C10H21O9P	Glycerophospholipids	315.0857	5.237	down	0.0472	up	0.0167
15	Diosgenin	C27H42O3	Prenol lipids	397.3107	8.221	up	0.0094	down	0.0201
16	Hippuric acid	C9H9NO3	Benzene and substituted derivatives	178.0515	5.402	down	0.0004	up	0.0273
17	4-Ethylbenzaldehyde	C9H10O	Benzene and substituted derivatives	135.0806	6.756	up	0.0398	down	0.0049
18	Eugenol	C10H12O2	Phenols	181.0875	5.723	up	0.0192	up	0.0000
19	tert-Butyl N-[1-(aminocarbonyl)-3-methylbutyl] carbamate	C11H22N2O3	Carboxylic acids and derivatives	231.1708	5.138	down	0.0012	up	0.0030
20	Creatinine	C4H7N3O	Carboxylic acids and derivatives	114.0664	1.316	up	0.0151	up	0.0053
21	Cyanuric acid	C3H3N3O3	Triaziness	128.0082	2.424	down	0.0183	up	0.0001
22	Neopterin	C9H11N5O4	Pteridines and derivatives	252.0731	1.389	up	0.0187	up	0.0013
23	Isophorone	C9H14O	Organooxygen compounds	139.1119	6.08	down	0.0470	up	0.0003
24	Verbascose	C30H52O26	Organooxygen compounds	827.2662	1.409	down	0.0250	up	0.0017
25	4-Methoxycinnamal-dehyde	C10H10O2	Cinnamaldehydes	163.0756	5.985	down	0.0053	down	0.0125
26	Aflatoxin G2	C17H14O7	Coumarins and derivatives	329.0698	6.403	down	0.0416	down	0.0027
27	Thymidine 5′-monophosphate	C10H15N2O8P	Pyrimidine nucleotides	321.0496	2.841	up	0.0148	up	0.0215
28	Normorphine	C16H17NO3	Morphinans	272.1286	5.987	down	0.0190	down	0.0008

## Data Availability

The 16S rRNA gene sequencing data generated and analyzed in this study were deposited in the China National Center for Bioinformation (CNCB, https://www.cncb.ac.cn) under accession number PRJCA038545.

## Supplementary Materials

The following supporting information can be downloaded at: https://www.mdpi.com/article/10.3390/ani15101409/s1, Table S1. Relative abundance of gut mcirobiota between four groups at genus level. Table S2. Relative abundance of gut mcirobiota between four groups at species level. Table S3. The intensity of neg-metabolites between four groups. Table S4. The intensity of pos-metabolites between four groups.

## Abbreviations

The following abbreviations are used in this article:

*B. licheniformis*	*Bacillus licheniformis*
*C. perfringens*	*Clostridium perfringens*
DAO	Diamine oxidase
D-LA	D-lactic acid
IL	Interleukin
TNF-α	Tumor necrosis factor-alpha
VFAs	Volatile fatty acids
SCFA	Short-chain fatty acid
HE	Hematoxylin and eosin
ASVs	Amplicon sequence variants
PCoA	Principal coordinates analysis
NMDS	Non-metric multidimensional scaling
KEGG	Kyoto Encyclopedia of Genes and Genomes
PLS-DA	Partial least squares discriminant analysis
PPP	Pentose phosphate pathway
PVDF	Polyvinylidene difluoride
Bcl-2	B-cell lymphoma-2
Bax	Bcl-2-associated X protein
